# Whole-exome sequencing in an extended family with myocardial infarction unmasks familial hypercholesterolemia

**DOI:** 10.1186/1471-2261-14-108

**Published:** 2014-08-26

**Authors:** Ingrid Brænne, Benedikt Reiz, Anja Medack, Mariana Kleinecke, Marcus Fischer, Salih Tuna, Christian Hengstenberg, Panos Deloukas, Jeanette Erdmann, Heribert Schunkert

**Affiliations:** Institute for Integrative and Experimental Genomics, University of Lübeck, 23562 Lübeck, Germany; DZHK (German Research Centre for Cardiovascular Research), partner site Hamburg/Lübeck/Kiel, 23562 Lübeck, Germany; Klinik und Poliklinik für Innere Medizin II, Universitätsklinikum Regensburg, 93053 Regensburg, Germany; Deutsches Herzzentrum München and Medizinische Klinik, Klinikum rechts der Isar, Technische Universität München, 80636 München, Germany; DZHK (German Centre for Cardiovascular Research), partner site Munich Heart Alliance, 80636 Munich, Germany; Wellcome Trust Sanger Institute, Wellcome Trust Genome Campus, Hinxton, CB10 1SA UK; William Harvey Research Institute, Barts and The London School of Medicine and Dentistry, Queen Mary University of London, London, UK; Princess Al-Jawhara Al-Brahim Centre of Excellence in Research of Hereditary Disorders (PACER-HD), King Abdulaziz University, Jeddah, 21589 Saudi Arabia

**Keywords:** Familial hypercholesterolemia, Myocardial infarction, Whole-exome sequencing

## Abstract

**Background:**

Familial hypercholesterolemia (FH) is an autosomal-dominant disease leading to markedly elevated low-density lipoprotein (LDL) cholesterol levels and increased risk for premature myocardial infarction (MI). Mutation carriers display variable LDL cholesterol levels, which may obscure the diagnosis. We examined by whole-exome sequencing a family in which multiple myocardial infarctions occurred at a young age with unclear etiology.

**Methods:**

Whole-exome sequencing of three affected family members, validation of the identified variant with Sanger-sequencing, and subsequent co-segregation analysis in the family.

**Results:**

The index patient (LDL cholesterol 188 mg/dL) was referred for molecular-genetic investigations. He had coronary artery bypass graft (CABG) at the age of 59 years; 12 out of 15 1st, 2nd and 3rd degree relatives were affected with coronary artery disease (CAD) and/or premature myocardial infarction (MI). We sequenced the whole-exome of the patient and two cousins with premature MI. After filtering, we were left with a potentially disease causing variant in the LDL receptor (*LDLR*) gene, which we validated by Sanger-sequencing (nucleotide substitution in the acceptor splice-site of exon 10, c.1359-1G > A). Sequencing of all family members available for genetic analysis revealed co-segregation of the variant with CAD (LOD 3.0) and increased LDLC (>190 mg/dL), following correction for statin treatment (LOD 4.3). Interestingly, mutation carriers presented with highly variable corrected (183–354 mg/dL) and on-treatment LDL levels (116–274 mg/dL) such that the diagnosis of FH in this family was made only after the molecular-genetic analysis.

**Conclusion:**

Even in families with unusual clustering of CAD FH remains to be underdiagnosed, which underscores the need for implementation of systematic screening programs. Whole-exome sequencing may facilitate identification of disease-causing variants in families with unclear etiology of MI and enable preventive treatment of mutation carriers in a more timely fashion.

## Background

Familial hypercholesterolemia (FH) may explain as much as 20 percent of familial cases with premature coronary artery disease (CAD) [1, 2]. Despite well-established criteria for clinical diagnosis and the proven benefits of medical treatment FH remains to be largely under-diagnosed and untreated [3]. In most European populations between 5-15% of cases are diagnosed [4, 5]. It has been considered that many FH cases are simply overlooked in the large number of CAD patients, a disease usually caused multifactorially by the interplay of common risk factors and common risk alleles [4, 6]. Moreover, with significant impact, the fact that nowadays most families are small may mask the inherited nature of the disease [7, 8].

In cases with suspected FH, family-based cascade screening by either clinical or molecular-genetic instruments is guideline recommended to unravel the autosomal-dominant mode of inheritance and to facilitate medical treatment at a young age (European Society of Cardiology [9], British National Institute for Clinical Excellence (NICE) [10], US Centres for Disease Control and Prevention [11]).

Yet, such testing either using lipid panels paired with clinical diagnostic criteria or molecular-genetic DNA sequencing of the currently known disease-causing genes is vastly under-used [4, 12, 13]. Exceptions are found in the Netherlands and Norway, where national screening programs for mutations in the disease-causing genes *LDLR, APOB* and *PCSK9* have been initiated [4, 14, 15].

Here, we report on an extended MI-family, which remained without diagnosis until whole-exome sequencing was performed. The index patient was referred to us because of premature MI (CABG at the age of 59) and positive family history for CAD (rather than for elevated LDL-cholesterol levels, LDL-C). His LDL-C was 188 mg/dL, which initially detracted from a clinical suspicion of FH. The family with 15 CAD cases (plus two that are related by marriage) and several untreated mutation carriers illustrates the need for a more systematic employment of molecular FH testing to increase the awareness and timely initiation of medical treatment.

## Methods

### MI family

**Figure 1:**
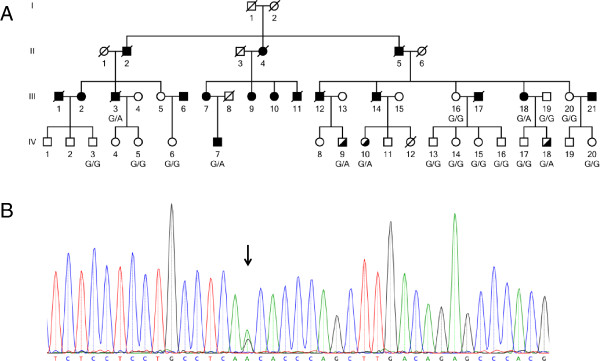
**Pedigree of the analyzed family and confirmation of mutation by Sanger sequencing. A)** The index patient is IV.7. The family members selected for whole-exome sequencing are III.3, III.18, and IV.7. Elevated LDL-C levels are shown as half filled symbols. Mutation carriers are shown as G/A and non-carriers as G/G. **B)** Confirmation of the nucleotide substitution in the acceptor splice-site of exon 10, c.1359-1G > A by Sanger sequencing. Shown is the sequence of the index patient IV.7. The black arrow points to the heterozygote position.

The index patient had suffered from CABG at the age of 59 years. Figure 1A depicts the pedigree of the MI family used for whole-exome sequencing. Clinical and angiographic characteristics of the family members available for analysis are described in detail in Table 1. All subjects analyzed in this study gave written informed consent before participating. The local Ethical Committee (University Regensburg, Germany) approved the study.

**Table 1:** **Clinical characteristics of family 6652**

Family ID	Pedigre ID	Sex ^$^	BMI (kg/m ^2^)	Age at first MI (years)	Age at first CAD (years)	LDL-C (mg/dL)	Statin therapy	Daily dose	Hyper-cholesterol-emia ^1#^	Hyper-tension ^2#^	Diabetes ^3#^	Smoking ^4#^
6652501	III.2	2	28.5		58	252	Atorvastatin	20	1	1	2	1
6652502	III.3	1	26.5		55	214	Atorvastatin	60	1	1	2	2
6652505	III.16	2	22.8			186			2	1	2	2
6652506	III.18	2	25.0		55	233	Atorvastatin	80	1	2	2	1
6652509	IV.6	2	26.4			184			2	2	2	1
6652511	III.6	1	25.9	44	67	189	Atorvastatin	10	1	1	2	1
6652512	III.20	1	30.5			136	Cerivastatin	0.3	1	1	2	2
6652513	IV.3	1	24.2			96			2	2	2	2
6652515	IV.5	2	23.4			171			2	2	2	1
**6652516**	**IV.7**	**1**	**25.2**		**59**	**188**	**Atorvastatin**	**40**	**1**	**2**	**2**	**2**
6652518	III.13	2	27.3			156			2	2	2	1
6652520	IV.9	1	22.5			142	Simvastatin	10	1		2	2
6652522	IV.13	1	29.4			166			2	2	2	1
6652523	IV.14	2	24.2			168			2	2	2	2
6652524	IV.15	2	23.5			130			2		2	2
6652525	IV.16	1	22.7			145			2	2	2	2
6652527	IV.20	2	23.4			143			2		2	1
6652531	IV.10	2	20.9			116	Atorvastatin	60	1	2	2	2
6652533	IV.12	2	21.3							2	2	2
6652534	III.19	1	23.1			115			2	1	2	2
6652535	IV.17	1	23.2			184			2	2	2	2
6652536	IV.18	1	22.8			274	Atorvastatin	20	1	2	2	2

$:1 = male, 2 = female; #: 1 = yes, 2 = no; ^1^Blood pressure >140/90 mm Hg; ^2^LDL cholesterol > 190 mg/dL or statin therapy; ^3^History of diabetes mellitus; ^4^History of smoking. Index patient IV.7 in bold.

### Exome sequencing

Whole-exome sequencing was performed as 54 bp paired-end runs on a Genome Analyzer IIx system (Illumina) after in-solution enrichment of exonic sequences (SureSelect Human All Exon 50 Mb kit, Agilent). Reads are mapped to the reference genome, duplicate fragments are marked using Picard and GATK is then used to recalibrate base qualities. We used the human genome assembly hg19 (GRCh37) as reference.

### Mutation validation

We annotated the single nucleotide variants (SNVs) with Annovar [16] using the UCSC database, Exome sequencing project and 1000 Genomes. Protein altering effect was predicted using SnpEFF [17] and avsift (Annovar). Mutation validation was performed by PCR and Sanger-sequencing of candidate gene region identified by whole-exome sequencing. After confirmation of the variant (Figure 1B), additional affected and unaffected family members were screened considering co-segregation. Primers used for mutation validation and PCR conditions are as following. A set of primers (Left Primer 5′-tgtaaaacgacggccagtGAGGCACTCTTGGTTCCATC-3′ and Right Primer 5′-caggaaacagctatgaccGTGGATACGCACCCATGAAC-3′) was chosen to amplify the region of the LDLR gene encompassing the c.1359-1G > A variant (562 bp length). Standard PCR was carried out in a 10 μl volume containing 10 ng genomic DNA, 5 pmol of each primer and 4 μl of Mastermix (VWR International GmbH, Darmstadt, Germany). Samples were processed in a Sensoquest labcycler with a standard touchdown PCR program (annealing temperature from 61°C-55°C).

## Results and discussion

We analyzed an extended German MI-family consisting of 53 members of whom 13 were affected with CAD and/or MI (Figure 1A). Of these, 18 family members were available for genetic analysis. Based on the phenotypic presentation in this family, we suspected an autosomal dominant mode of inheritance. Therefore, we decided to carry out whole-exome sequencing of three affected family members to unravel the genetic cause of CAD/MI in this family.

The amount of disease-unspecific variants present in the downstream analysis of whole-exome sequencing is largely influenced by the subset of family members that are selected for the initial analysis [18, 19]. To optimize the variant search, one strategy is to select distantly related family members. Whereas siblings share 50% of the genetic variants, cousins share only 12.5% [20]. Hence, we selected cousins instead of siblings to reduce the number of disease-unspecific variants and to increase the chance for identifying the truly disease-causing variants (Figure 1A; III.3, III.18, and IV.7) [21, 22].

To identify the causal variant in this family, we based our filtering on three assumptions: (1) the variant is inherited in an autosomal dominant mode of inheritance; (2) the variant is very rare (<0.1%) in the general population; and (3) most affected family members carry the variant (high penetrance).

The sequencing was performed on a Genome Analyzer llx system (Illumina) after in-solution enrichment of exonic sequences. The exome sequencing revealed an average read depth of 155 with minimum 84% of the target regions covered at least 20X.

Whole-exome sequencing revealed around 39,000 variants in total and approximately 20,000 located in exonic regions. We filtered the variants using the frequencies stated in 1000 Genomes (1 kG, Annovar version 1000g2012apr), Exome Sequencing Project (ESP, Annovar version 6500) and in internal exome data (8 samples sequenced with the same platform). Around 1,800 variants were found with a frequency less than 0.1% in ESP and 1 kG. In a next filtering step, we kept only variants that were conserved and outside regions of segmental duplication, leaving ~825 variants. To identify variants with potentially severe function-altering effects, we filtered all variants based on avsift score >0.05 and "HIGH" effect predicted using Annovar and SnpEFF. These variants are either amino acid changes, stop gain, stop loss or splice-site mutations (more details can be found in Table 2). Only one variant, a G > A substitution in the acceptor splice-site of exon 10 in the *LDLR* gene (IVS9-1G > A or c.1359-1G > A), was shared by the three sequenced family members and was validated by Sanger-sequencing.

**Table 2:** **Bioinformatic filtering of the family members that were exome sequenced**

Filter	III.3	III.18	IV.7	Mean
Total aligned variants	38,702	38,408	38,775	38,628
Variants (exome)	20,344	20,387	20,173	20,301
Not in internal controls	1,995	2,123	2,097	2,072
Variants with frequency <0.1% in 1000G and ESP	1,643	1,885	1,864	1,797
Conserved and no segmental duplication	713	900	862	825
snpEFF = HIGH and AVSIFT <0.05	7	18	19	15
Shared variants				1

The data was annotated using annovar [16] (Version August 2013). Functional prediction was performed with snpEFF [17] (build 2014-01-16) and AVSIFT (Annovar).

This G > A substitution results in the loss of a splice-site with a predicted splice-site score of -4.2 for the variant versus 6.8 for the reference allele [ http://rulai.cshl.edu]. We genotyped this variant in all family members available for genetic analysis to check for co-segregation with the disease. There were 18 (one related by marriage) family members available for genotyping. Of these, three were affected and these carried the *LDLR*-variant. Of the 14 unaffected family members, which were not related by marriage, three carried the variant. The three unaffected mutation carriers were, at the time included in this analysis, 48, 54 and 54 years old. Since MI is a late-onset disease, we expect the disease to occur late in life. Hence, the affection status regarding MI must be considered unknown at this point.

Since the 1990s, it is well established that mutations in the *LDLR* gene are the major cause for familial hypercholesterolemia [1, 23]. Around 90% of all known disease-causing mutations (more than 1,800 mutations are listed in HGMD (version 2013.4)) for FH are found in the *LDLR* gene [3, 24, 25]. FH is also caused by mutations in the *APOB* and *PCSK9* genes, with frequencies of around 5% and ~1% respectively [4, 26].

We compared the LDL-C levels of the family members that carry the variant with those that do not carry the variant. The results showed that the variant co-segregates with increased LDL-C levels (LOD 4.3). The average LDL-C levels were significantly increased in mutation carriers compared with non-mutation carriers (279 mg/dL versus 155 mg/dL). However, the plasma levels were highly variable ranging from 183 mg/dL to 354 mg/dL; this fact misled the clinical diagnosis in the index patient (188, corrected for statin use 279 mg/dL). To estimate the functional implication of the identified variant, we systematically searched the literature. This revealed that the LDLR variant has been previously described as disease-causing in families with FH [27, 28].

This variant is reported to originate from the south of the Netherlands and to significantly increase the LDL-C levels and hence cause a higher risk of premature CVD. The risk increase was found to be 15.95% for mutation carriers compared to unaffected relatives [29]. In our analyzed family, all but one mutation carrier have LDL-C levels above 190 mg/dL. This result clearly underlines the previous reported significant risk increase.

The variant cause an abnormally spliced transcript, which results in a deletion of 7 bp and a frame shift with premature termination [30]. The deletion affects the normal acceptor splice site in the 9th intron. This leads to a cryptic splice site that consist of the 6th and 7th nucleotide of exon 10.

The variant is likely to cause haploinsufficiency because of the truncated transcript and hence nonsense-mediated decay. However, even a protein product would lead to a loss-of-function of the receptor since several important domains of the receptor would be missing, such as the crucial transmembrane region, the C-terminal part of the epidermal growth factor precursor homology domain, the O-linked sugar domain, the membrane-spanning domain, and the cytoplasmic domain. Other variants lacking these domains have been reported to have reduced activity (around 2%) and are classified as null allele (class 1) mutations [31]. These mutations produce normal mRNA transcripts, however, in reduced concentration [32], which explains the increased risk of FH caused by this mutation.

With FH as the underlying cause of MI in this family, we could have anticipated a mutation in the LDLR gene. However, the index patient was referred to us and included in our study with the diagnosis of premature MI/CABG and positive family history for CAD.

The varying LDL-C levels in this family had initially hindered the diagnosis of FH. It is, however, well known, that the LDL-C levels may vary remarkably even among individuals with the same mutation. One explanation for this is that other modifying factors are present [31]. One such mutation was described by Hobbs et al. [31]. They reported a family in which one-third of the mutation carriers have LDL concentrations below the 90th percentile. They suggested the existence of a dominant gene that suppresses the effect of the LDL receptor mutation. Hence, the fact that one of our mutation carriers have LDL-C levels below 190 mg/dL might have similar explanation and demonstrate the complexity of even expected monogenic disease variants.

The result of this study underlines the power of whole-exome sequencing approaches to identify disease-causing variants. Whole-exome sequencing is a promising tool to identify the cause of a disease in families with unclear etiology of MI.

## Conclusion

We strongly suggest that whole-exome sequencing can be used for stratified medicine. If we know the disease-causing variant in affected families, we can enable specific preventive treatment of so-far unaffected mutation carriers and this may prevent or delay the onset of the disease. In addition, having overseen FH in the family at hand, we believe that the systematic sequencing of *LDLR* pathway genes may accelerate the diagnostic work-up.
